# The impact of neuroradiology collaboration in head and neck cancer radiotherapy peer review

**DOI:** 10.1259/bjr.20210238

**Published:** 2022-12-19

**Authors:** Elinor R Gatfield, Richard J Benson, Rashmi Jadon, Tilak Das, Gillian C Barnett

**Affiliations:** 1 Department of Clinical Oncology, Addenbrooke’s Hospital, Hills Road, Cambridge, UK; 2 Department of Radiology, Addenbrooke’s Hospital, Hills Road, Cambridge, UK; 3 Department of Oncology, University of Cambridge, Cambridge Biomedical Campus, Addenbrooke’s Hospital, Hills Road, Cambridge, UK

## Abstract

**Objective:**

To quantify the impact of neuroradiologist presence on head and neck cancer (HNC) radiotherapy peer review (PR) changes.

**Methods:**

Prospective data were collected from HNC radiotherapy PR meetings; major, minor, and organ at risk (OAR) changes recorded. Differences in changes made with a neuroradiologist present were determined. χ^2^ tests of statistical significance were performed. Multivariate logistic regression identified potential predictors of changes.

**Results:**

Prospective PR was performed in 125/160 (78%) patients undergoing radical (chemo)radiotherapy for HNC between October 2018 and September 2019. Full PR documentation was available for 120/160 meetings (75%), with a neuroradiologist present in 53/120 (44%). Overall, 51/120 (42.5%) had changes made to target volumes or OARs. When a neuroradiologist was present, 29/53 (55%) of plans had changes made, compared to 22/67 (33%) in their absence. On multivariate analysis, neuroradiologist presence significantly influenced any changes made during the PR meetings (OR 2.59; 95% CI 1.05–6.43; *p* = 0.039).

**Conclusion:**

Neuroradiologist presence at PR meetings significantly influences changes made to HNC contouring, likely improving consistency and enhancing quality assurance.

**Advances in knowledge:**

This is the first published UK series demonstrating that a collaborative approach between radiology and oncology in PR meetings is significant in leading to contour changes for HNC.

## Introduction

It has been recognised for over a decade that the quality of contouring for head and neck cancer (HNC) affects clinical outcomes with respect to both treatment failure and normal tissue toxicity.^1,2^ Interclinician contour outlining varies even in standardised HNC trials,^2^ possibly due to the difficulties in interpreting complex anatomy, hence the importance of peer review. Evidence suggests that the vast majority of changes made to target volume and organ at risk (OAR) definition at peer review can be detected at the post-contouring stage.^3,4^

The Royal College of Radiologists (RCR) guidance (2017) recommends prospective contour peer review for quality assurance, stating that attendance of a radiologist “may be useful”.^5^ Two prospective series of skull base, central nervous system and radically treated HNC patients with neuroradiologist review led to changes in 47 and 55% of plans,^6,7^ with 32% classified as major and 14% minor.^6^ Absolute volume change for gross tumour volume (GTV) was 14.88 mm^3^ (19.75%) and clinical target volume (CTV) was 14.63 mm^3^ (21.83%).^7^ Small changes to target volumes were sometimes highly clinically significant, *e.g.* lymph node involvement, osseous infiltration, or perineural extension.^7^

## Methods

Here, we discuss data collected prospectively from scheduled weekly peer review meetings at the post-contouring stage, prior to treatment planning, to quantify the impact of the presence of a neuroradiologist on peer review changes for radically treated HNC radiotherapy plans. Major, minor, and OAR changes were recorded as well as any significant discussion including dose, concurrent agents, fusion, choice of imaging sequence, and choice of nodal levels. A major change was defined as an alteration to the GTV or high-dose CTV and a minor change was defined as an alteration to the elective-dose CTV.^5,8^ For these current series, if both major and minor changes occurred following peer review, this was recorded as a major change. Differences in the changes made in the presence or absence of a neuroradiologist were documented and χ^2^ tests were performed to assess statistical significance. Multivariate logistic regression was used to identify potential predictors of any change, a major change or a change to the GTV. A *p* value <0.05 was considered statistically significant.

## Results

All patients undergoing radical (chemo)radiotherapy for HNC between October 2018 and September 2019 at Addenbrooke’s Hospital, Cambridge, were included (*n* = 171). 11 patients with early larynx cancer were excluded. Prospective peer review was performed for 125 (78%) patients and full peer review documentation was available for 120 (75%). Of the 120 patients with completed documentation, a neuroradiologist was present at 53 (44%) peer reviews. Overall, 51 (42.5%) plans had a change made to any of the target volumes or OARs. Differences in changes made with or without the presence of a neuroradiologist are summarised in Table 1. Changes were only made by agreement, with the prescribing oncologist making the final decision. On multivariate analysis, including treating consultant and diagnosis, the presence of a neuroradiologist significantly influenced any change made during the peer review meetings (OR 2.59; 95% CI 1.05–6.43; *p* = 0.039), a major change (OR 4.04; 95% CI 1.51–10.8; *p* = 0.005) or a change to the GTV (OR 9.72; 95% CI 2.56–36.9; *p* = 0.001).

**Table 1.** Differences in the changes made at peer review to head and neck cancer radiotherapy plans post-contouring, determined by the presence or absence of a neuroradiologist

	Neuroradiologist present (*n* = 53)	Neuroradiologist absent (*n* = 67)	*p*-value
Any change	29 (55%)	22 (33%)	0.016
Any major	27 (53%)	16 (24%)	0.002
Major – GTV	20 (38%)	6 (9%)	<0.001
Major – high-dose CTV	26 (51%)	16 (24%)	0.004
Minor	6 (11%)	10 (15%)	0.564
OAR	5 (9%)	1 (1%)	0.047
Discussions	39 (75%)	33 (49%)	0.007

GTV, gross tumour volume; CTV, clinical target volume; OAR, organ at risk

Significant discussions were documented, including dose, concurrent agents, fusion, choice of imaging sequence, and choice of nodal levels

## Discussion

Table 2 compares peer review changes for HNC patients at different UK cancer centres. Although there are differences between locations, possibly attributable to the mix of cases, it is notable that the percentage of peer review changes is higher in the presence of a neuroradiologist, consistent with the published series from Jacksonville and San Francisco, USA.^6,7^ It is worth noting that the definition of major and minor changes in the current series differs from the Birmingham cohort, where significant changes included those with a high risk of geographical miss or extensive unnecessary impact on critical OARs or normal tissues, and minor changes were those when the original would have been acceptable.^10^ The criteria for major and minor changes in the current series was based on RCR guidance.^5^ This may account for the differences observed, as major changes in the current series could have been considered minor as per the definitions used by Fong et al.^10^

**Table 2.** Summary comparison of selected UK cancer centres reporting head and neck peer review changes

	Number of cases	Unchanged	Major change	Minor change
Exeter, Taunton and Torbay^9^	129 (radical/adjuvant and palliative)	88%	3%	9%
Birmingham^10^	62 (radical/adjuvant)	61%	13%	26%
Leeds^8^	307 (radical/adjuvant)	86%	9%	5%
Current series (neuroradiologist absent)	67 (radical/adjuvant)	67%	24%	9%
**Current series (neuroradiologist present**)	**53 (radical/adjuvant**)	**45%**	**51%**	**4%**

In this current series, data on the median volume change or a range were not collected. It has previously been shown that measuring volumes or conformity indices, are not a reliable alternative to expert assessment, as they correlate poorly. For example, in the EMBRACE-II study, a Jaccard Conformity Index cut-off of 0.7 would have identified 87% contours that failed expert assessment, but also excluded 54% of passing contours.^11^ Moreover, the magnitude in the change in volume does not necessarily reflect the potential clinical consequences.

Although the work-up for HNC radiotherapy is complex and time-consuming, these data show that the availability of a neuroradiologist to join scheduled peer review meetings is significant in leading to contour changes, without adding a delay to treatment pathways. This collaborative approach between radiology and oncology therefore represents a more effective use of diagnostic and planning imaging to enhance quality assurance, with the aim of giving HNC patients the best possible outcomes following radical radiotherapy.
